# 

**Published:** 1916-08

**Authors:** 


PUBLISHED MONTHLY. ATLANTA SUBSCRIPTION $1.00

### ATLANTA JOURNAL-RECORD OF MEDICINE

Successor to { Atlanta Medical and Surgical Journal, Established 1855, and Southern Medical Record, Established 1870. } 63rd Year

Vol. LXIII ATLANTA, GA., AUGUST, 1916 No. 5

